# Ex Vivo High Salt Activated Tumor-Primed CD4+T Lymphocytes Exert a Potent Anti-Cancer Response

**DOI:** 10.3390/cancers13071690

**Published:** 2021-04-02

**Authors:** Venkataswarup Tiriveedhi, Michael T. Ivy, Elbert L. Myles, Roy Zent, Jeffrey C. Rathmell, Jens Titze

**Affiliations:** 1 Department of Biological Sciences, Tennessee State University, 3500 John A Merritt Blvd, Nashville, TN 37209, USA; mivy@tnstate.edu (M.T.I.); emyles@tnstate.edu (E.L.M.); 2 Division of Pharmacology, Vanderbilt University, Nashville, TN 37212, USA; 3 Division of Nephrology and Hypertension, Department of Medicine, Vanderbilt University Medical Center, Nashville, TN 37232, USA; roy.zent@vumc.org; 4 Department Pathology, Microbiology and Immunology, Vanderbilt University Medical Center North, Nashville, TN 37232, USA; jeff.rathmell@vumc.org; 5 Program in Cardiovascular and Metabolic Disorders, Duke-NUS Medical School, Singapore 169857, Singapore; jens.titze@duke-nus.edu.sg; 6 Division of Nephrology, Duke University School of Medicine, 2 Genome Court, Durham, NC 27710, USA

**Keywords:** breast cancer, immunotherapy, T-helper cells, cytokines, cancer biology

## Abstract

**Simple Summary:**

Cell based immunotherapy is rapidly emerging as a promising cancer treatment. Salt (sodium chloride) treatment to immune cell cultures is known to induce inflammatory activation. In our current study, we analyzed the anti-cancer ability of salt treatment on immune cells outside the host followed by reinfusion into the host. Using a pre-clinical breast cancer model, we demonstrated that external salt treatment on T-cell subset of immune cells produced a viable anti-cancer response, which may have future human clinical application.

**Abstract:**

Cell based immunotherapy is rapidly emerging as a promising cancer treatment. A modest increase in salt (sodium chloride) concentration in immune cell cultures is known to induce inflammatory phenotypic differentiation. In our current study, we analyzed the ability of salt treatment to induce ex vivo expansion of tumor-primed CD4 (cluster of differentiation 4)+T cells to an effector phenotype. CD4+T cells were isolated using immunomagnetic beads from draining lymph nodes and spleens from tumor bearing C57Bl/6 mice, 28 days post-injection of Py230 syngeneic breast cancer cells. CD4+T cells from non-tumor bearing mice were isolated from splenocytes of 12-week-old C57Bl/6 mice. These CD4+T cells were expanded ex vivo with five stimulation cycles, and each cycle comprised of treatment with high salt (Δ0.035 M NaCl) or equimolar mannitol controls along with anti-CD3/CD28 monoclonal antibodies for the first 3 days, followed by the addition of interleukin (IL)-2/IL-7 cytokines and heat killed Py230 for 4 days. Ex vivo high salt treatment induced a two-fold higher Th1 (T helper type 1) expansion and four-fold higher Th17 expansion compared to equimolar mannitol treatment. Importantly, the high salt expanded CD4+T cells retained tumor-specificity, as demonstrated by higher in vitro cytotoxicity against Py230 breast cancer cells and reduced in vivo syngeneic tumor growth. Metabolic studies revealed that high salt treatment enhanced the glycolytic reserve and basal mitochondrial oxidation of CD4+T cells, suggesting a role of high salt in enhanced pro-growth anabolic metabolism needed for inflammatory differentiation. Mechanistic studies demonstrated that the high salt induced switch to the effector phenotype was mediated by tonicity-dependent transcription factor, TonEBP/NFAT5. Using a transgenic murine model, we demonstrated that CD4 specific TonEBP/NFAT5 knock out (CD4^cre/cre^NFAT5^flox/flox^) abrogated the induction of the effector phenotype and anti-tumor efficiency of CD4+T cells following high salt treatment. Taken together, our data suggest that high salt-mediated ex vivo expansion of tumor-primed CD4+T cells could induce effective tumor specific anti-cancer responses, which may have a novel cell-based cancer immunotherapeutic application.

## 1. Introduction

Adoptive T cell transfer (ATCT) with the reinfusion of isolated and ex vivo activated T lymphocytes is emerging as a novel cell-based cancer immunotherapeutic strategy [1]. Tumor-specific T cells are highly enriched within tumors and in the draining lymph nodes [2]. De novo expansion of naïve T cells might limit antigen specificity and anti-tumor efficiency [3]. However, tumor-primed T-cells retain their T-cell receptor (TCR) specificity in progeny cells, making it a feasible therapeutic option [4]. ATCT has a unique advantage over other active immunization strategies in that it does not require in vivo activation and expansion of antigen specific T cell responses by a cancer patient with an otherwise weak immune system. Specific in vitro immune depletion strategies prior to infusion have met with limited success due to CD4 (cluster of differentiation)+T cell plasticity [5,6,7,8]. The optimal protocols for ex vivo T cell activation to evoke a favorable effector anti-tumor response for clinical application are still unclear. 

Previous adoptive immunotherapy studies have been based on the co-application of systemic interleukin (IL)-2 administration along with T-cell transfer to enhance in vivo T cell effector functionality [9,10,11]. However, this approach has shown a restricted infiltration of transferred T cells to metastatic sites and the proliferation of immunosuppressive regulatory CD4+T cell (Treg) phonotype, along with a lack of general agreement on IL-2 dosing [12]. Similar challenges have been noted on the application of other cytokines such as IL-15 [13]. Moreover, these cytokines were needed in high concentrations and that too only transiently in the local microenvironment during antigen presentation, T-cell activation and clonal proliferation, thus rendering the continuous systemic administration of cytokines amenable to unnecessary off-target side-effects such as capillary leak syndrome and cytokine storm [14,15]. Significant scientific work over the recent years has focused on evaluating the anti-cancer ability of adoptively transferred T cells. Preliminary studies using ex vivo IL-2 expansion of tumor infiltrating lymphocytes (TILs) and peripheral blood immune cells followed by autologous transfer into the cancer patients demonstrated the expansion of these cells with weak antigen specificity and poor anti-tumor effect [16,17]. Further, adverse immunosuppressive tumor microenvironment limits the activation of these transferred TILs. Studies with systemic lymphodepletion prior to ATCT have resulted in mixed outcomes with enhanced systemic inflammatory side-effects. A lack of in vivo activation and the weak tumor-specificity of adoptively transferred CD4+T cells remain a major challenge in the long term success of ATCT. 

Salt (sodium chloride) has been shown to activate CD4+T cells and induce effector phenotypic differentiation. In various hypertensive models, a 35–40 mM higher circulating sodium concentration induced by dietary high salt was shown to induce inflammatory T helper (Th)17 activation of T-cells, resulting in end-organ damage [18]. Similarly, in autoimmune models such as experimental autoimmune encephalitis (EAE) and graft-versus host disease (GvHD) murine models, a high salt diet induced inflammatory activation and effector CD4+T cell responses to self-antigens, and at the same time decreased immunosuppressive Treg responses [19,20,21,22]. Further, tonicity-sensitive transcription factor (NFAT5, nuclear factor of activated T cells, also called TonEBP) is known to play a significant role in the salt-mediated exaggeration of inflammatory immune responses [23]. The activation of effector CD4+T cells along with the suppression of Treg responses would be central to the long-term success of cancer immunotherapy. However, a generalized recommendation of a high salt diet to induce anti-cancer inflammatory CD4+T cell responses in cancer patients would be a non-viable option. In view of this limitation, using a murine breast tumor model, we tested a hypothesis that ex vivo high salt activation of CD4+T cells from tumor bearing mice followed by the adoptive transfer of these T cells would exert effective anti-tumor responses.

## 2. Materials and Methods

### 2.1. Animals and Cell Lines

Female C57Bl/6 mice were purchased from the Jackson Laboratories (Maine, ME, USA). Mice were housed in specific pathogen-free facilities in ventilated cages with at most 5 animals per cage, provided *ad libitum* food and water, and used at 8–12 weeks of age. All protocols followed the guidelines approved by the Vanderbilt Institutional Animal Studies Committee (Protocol#M1600078-00-AN2). Transgenic mice with CD4-specific knock-out of TonEBP (CD4^cre/cre^TonEBP^flox/flox^C57Bl/6) were generated in Titze laboratory and kindly provided to us. Tail-snip genotyping was performed by PCR to test for the transgenic knock-out. The Py230 syngeneic murine breast cancer cells were trypsinized and washed with PBS (phosphate-buffered saline) before intra-mammary injection (0.75 × 10^5^ cells/mice) into the flank of the mice. The tumor volume was calculated using the formula V = (W^(2) × L)/2 from caliper measurements, where V is tumor volume, W is tumor width and L is tumor length [24,25]. Single-cell suspensions were prepared from explanted murine breast tumors by digestion with a mixture of 0.1% collagenase type IV, 0.01% DNase I, and 2.5 U/mL hyaluronidase type V in HBSS (Hank’s balanced salt solution) for 2 h at room temperature. These cells were filtered through a 100 μm nylon mesh, washed, and resuspended in HBSS for further studies. The Py230 murine cancer cells were obtained from ATCC (Manassas, VA, USA). Appropriate mycoplasma free testing was performed on cell cultures to prevent contamination. The cells were cultured in RPMI1640 media in 5% CO_2_ incubator along with the media supplements such as fetal bovine serum, penicillin/streptomycin, fungizone, HEPES (4-(2-hydroxyethyl)-1-piperazineethanesulfonic acid ) and glutamine, as recommended by the manufacturer and as previously described. All chemicals unless mentioned were obtained either from Sigma-Aldrich (St. Louis, MO, USA) or Fisher Scientific (Waltham, MA, USA).

### 2.2. Isolation and Ex Vivo Activation of CD4+ T-cell 

The breast tumor bearing C57Bl/6 mice were sacrificed on day 28 post-injection of breast cancer cells to obtain draining lymph nodes (DLNs) and spleens. DLNs were physically teased from the surrounding supportive tissue with a 20-guage needle. Similarly, spleens were also harvested from non-tumor bearing mice. Single cell preparation was prepared from these harvested tissues. CD4+T cells were isolated using immunomagnetic beads (MACS beads, Miltenyi Biotec, Sunnyvale, CA, USA), first by depletion of CD8+T cells and then with positive selection with CD4+T cells. The purified CD4+T cells were suspended in complete medium (CM). These isolated cells (initial cell count of 0.3 million CD4+T cells) were expanded ex vivo for five stimulation cycles. Each stimulation cycle lasted for 7 days, consisting of treatment with 35 mM NaCl, anti-CD3 monoclonal antibody (mAb, 2 μg/mL) CD28 mAb (2 μg/mL) for 3 days, followed by addition of IL-7 (10 ng/mL), IL-2 (20 ng/mL) and heat killed Py230 cells for the next 4 days. An equimolar (35 mM) mannitol was used instead of NaCl as osmotic control in our experiments. Heat killed Py230 cells (at 95 °C for 15 min) were added to the cultures to obtain a final CD4+T cell to (heat killed) cancer cell ratio of 1:10. Following each stimulation, activated cells were washed and resuspended at 0.5 × 10^5^/mL in complete media to repeat the next stimulation cycle. 

### 2.3. FACS Analysis and Intracellular Staining

Fluorophore-conjugated antibodies for CD4, Tbet, RORγT, FoxP3, IFNγ, IL-17 and isotype-matched rat Ig, with appropriate fluorophore choices based on specific channel needs (FL1: FITC, Alexa 488; FL2: PE, FL3: PerCP, and FL4: APC, Cy5), were purchased from BD Biosciences (San Diego, CA, USA). The CD4+T cells were appropriately purified, washed and resuspended in the flow buffer and pretreated with FcR block, followed by staining for 30 min with a mixture of conjugated-primary antibodies. The stained cells were fixed with 2% paraformaldehyde for 20 min before flow analysis. For intracellular staining, appropriate permeabilization buffers were used as per manufacturer’s recommendation. For in vivo trafficking studies, CD4+T cells were labeled with Cell Trace^TM^ Violet (CTV, Fisher Scientific, Hampton, NH, USA) as per manufacturer’s recommendation. For tumor localization, CD4+T cells were isolated and purified from tumors in single cell suspension as mentioned above. The frequency of CD4+T cell phenotypes was analyzed by flow using appropriate conjugated antibodies (noted above). For flow cytometer-based cytotoxicity assay, target cells (cancer cells) were labelled with cell membrane fluorophore PHK26 and cell death was analyzed by double positive staining for PHK26 and 7-AAD. Cells were analyzed using a FACS Calibur^TM^/LSRII flow cytometer (Becton-Dickinson, Franklin Lakes, NJ, USA), and cell sorting was performed using a Vantage cell sorter (Becton-Dickinson). Data were analyzed using BD FACSDiva software (BD Biosciences, San Jose, CA, USA). Gates were set according to isotype controls.

### 2.4. Adoptive Immunotherapy

Therapeutic efficacy of activated T cells was assessed in Py230 murine breast tumor models in C57Bl/6 mice. We administered 5 × 10^6^ CD4+ T-cells positively selected and activated from three stimulation cycles (mentioned above) for injection into 21 day tumor bearing mice. The animals were followed up for 45 days to perform tumor volume studies. 

### 2.5. Quantitative Real Time Polymerase Chain Reaction 

Expression profiles of genes at mRNA (messenger ribonucleic acid) level in the murine breast tumors were analyzed using the TaqMan FAM-labeled RT-PCR primers for NFAT5 (Mm00467257_m1), GADPH (Mm00467257_m1), and Actin (Mm02619580_g1), obtained from Applied Biosystems/Thermo Fisher Scientific (Grand Island, NY, USA) as per the manufacturer’s recommendation [26]. Briefly, total RNA was extracted from 10^6^ cells using TRIzol reagent (Sigma-Aldrich, St. Louis, MO, USA) and analyzed as mentioned previously. RNA samples were quantified by absorbance at 260 nm. The RNA was reverse-transcribed and RT-PCR (real time PCR) was performed in a final reaction volume of 20 μL using BioRad CFX96 (Hercules, CA, USA). Each sample was analyzed in triplicate. Cycling conditions consisted of an initial denaturation of 95 °C for 15 min, followed by 40 cycles of 95 °C for 30 s, followed by 61 °C for 1 min.

### 2.6. Western Blot

Total proteins were extracted from cells with lysis buffer. The supernatant was collected from cell lysate after running on a centrifuge at 13,500× *g* rpm for 20 min at 4 °C, and as previously described [27]. Protein concentration was determined with a Bradford assay kit from Bio-Rad (Philadelphia, PA, USA). Total proteins were separated on a 4–12% sodium dodecyl sulfate-polyacrylamide gradient gel and electrophoretically transferred onto a nitrocellulose membrane. The membranes were blocked overnight at 4 °C in Tris-buffered saline with 0.05% Tween 20 (5% nonfat milk in 10 mM Tris-HCl 100 mM NaCl 0.1% Tween 20, pH 7.4). The membranes were incubated first with antibodies (Abs) specific for total and phosphorylated forms at room temperature with primary Abs diluted 1 in 1000 in blocking buffer for 2 h, and then with a horseradish peroxide-conjugated secondary IgG mAb diluted 1 in 5000 for 1 h. All primary and secondary Abs were obtained from Santa Cruz Biotech (Dallas, TX, USA) unless indicated otherwise. The following specific primary antibodies to NFAT-5 (sc-398171) and GADPH (sc-32233) were utilized. The membrane was developed using the chemiluminescence kit (Millipore) and analyzed using Bio-Rad Universal Hood II (Hercules, CA, USA). Morphometric analysis was performed using the software provided by the company.

### 2.7. Cytokine Assay 

The secretory extracellular cytokines (IL-10, interferon [IFN]γ, tumor necrosis factor [TNF]α, IL-17) in the supernatant from cells treated under various assay conditions were quantitated by ELISA as per the manufacturer’s protocol (Life Technologies, Grand Island, NY, USA) [28]. The protein supernatant was diluted 1:500 and quantified with a standard curve using the manufacturer provided standards. Detection at 450 nm was performed using an EMax Plus spectrophotometer and data analysis was carried out using software provided by the manufacturer (Molecular Devices, Sunnyvale, CA, USA).

### 2.8. Cytotoxicity Assay 

The cytotoxic efficiency of CD4+T cells was investigated by their ability to lyse the target breast cancer cells by non-irradiative LDH (lactate dehydrogenase) release assay (Promega, Madison, WI, USA) [29]. The Py230 murine breast cancer cells (1 × 10^4^ cells, referred to as target cells) incubated at 37 °C in 200 µL of complete medium were plated in quadruplicate cultures in round bottom 96-well plates in the presence of varying numbers of activated CD4+T cells (referred to as effector cells), with an effector to target (E:T) ratio maintained at 20:1. The percentage specific lysis was calculated as follows: [(experimental LDH release − spontaneous LDH release)/(maximum LDH release − spontaneous LDH release)] × 100.

### 2.9. Cell Proliferation Assay

The viability of ex vivo expanded lymphocytes was first verified by trypan blue dye exclusion (Sigma Aldrich, St. Louis, MO, USA), followed by propidium iodide and annexin V staining (Sigma Aldrich). The quantitative cell proliferation was performed and reported based on BrdU cell proliferation assay (Cell Signaling Technology, Danvers, MA, USA) as per manufacturer’s protocol. 

### 2.10. Metabolic Assays

The metabolic activity of CD4+T cells was studied by analyzing the extracellular acidification rate (ECAR) and oxygen consumption rate (OCR) using the Seahorse XF24 Extracellular Flux Analyzer (Agilent, Santa Clara, CA, USA) [30]. Glycolytic capacity was defined as the difference between ECAR following the injection of oligomycin (1 μM) and the baseline ECAR reading (CD4+T cells cultured in basal DMEM (Dulbecco’s modified Eagle medium) with no added glucose or glutamine). Apparent glycolytic reserve was defined as the difference in ECAR between the glucose and oligomycin injections. Apparent respiratory reserve capacity was defined as the percentage increase in OCR between the initial baseline measurement (CD4+T cells cultured in basal DMEM with 25 mM glucose added) and the injection of 500 nM of the ionophore FCCP (Carbonylcyanide-p-trifluoromethoxyphenylhydrazone, Seahorse Bioscience, North Billerica, MA, USA), an uncoupler of oxidative phosphorylation and electron transport. The 2DG (2-deoxy D-glucose, 250 μM) was used for inhibition of glycolysis, and rotenone (5 nM)/antimycin A (10 nM) was used for inhibition of mitochondrial complexes I and III. OCR and ECAR values were normalized to cell number in each well.

### 2.11. Statistical Analysis

Data were expressed as the mean ± SEM (standard error of mean). Significant differences between groups were assessed using Tukey HSD pair-wise comparisons for two groups and one-way ANOVA for multiple comparisons. A *p*-value of <0.05 was considered significant. All data analysis was obtained using Origin 6 software (Origin Labs, Northampton, MA, USA) or SPSS software, version 21 (IBM corporation, Armonk, NY, USA).

## 3. Results

### 3.1. Ex Vivo High Salt Treatment Induces CD4+T cell Expansion and Effector Differentiation

**Figure 1:**
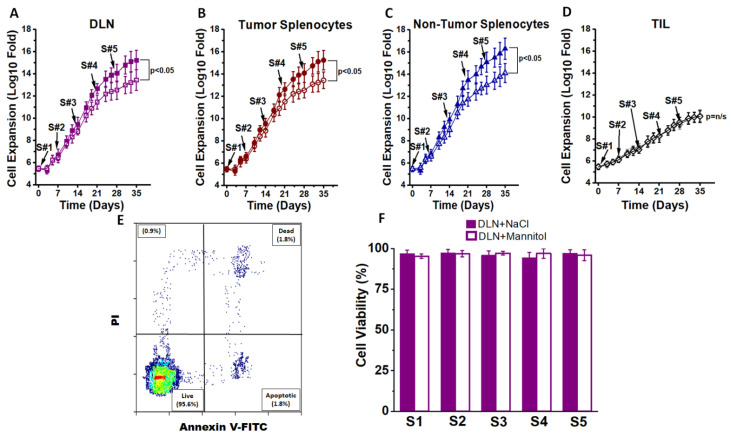
Ex vivo expansion of CD4+T cells. (**A**) The CD4+T cells obtained from draining lymph nodes (DLNs) of day 28 Py230-othrotopic syngeneic breast tumor bearing mice were expanded in the presence of high salt culture treatment (∆0.035 M NaCl, filled boxes) or with control equimolar mannitol (0.035 M mannitol, open boxes). CD4+T cells were subjected to five stimulation cycles (S1–S5), with each cycle lasting for 7 days and a total of 35 days. Stimulation cycle details including other chemicals used in the cell culture are provided in the “Materials and Methods” and “Results” sections. (**B**) The CD4+T cells obtained from the spleens of day 28 tumor bearing mice were expanded in the presence of high salt culture treatment (filled circles) or with control equimolar mannitol (open circles). (**C**) The CD4+T cells obtained from the spleens of 12-week-old non-tumor bearing mice were expanded in the presence of high salt culture treatment (filled triangles) or with control equimolar mannitol (open triangles). (**D**) CD4+Tumor infiltrating lymphocytes (TILs) were isolated by magnetic bead separation and stimulated for 5 cycles with either high salt (filled grey diamond) or mannitol (open diamond). (**E**) Cell viability test for staining with propidium iodide and annexin V staining. (**F**) Cell viability reported at the end of each 7 day stimulation cycle on CD4+T cells obtained from DLNs. A similar >95% viability was noted on CD4+T cells obtained from tumor splenocytes, non-tumor splenocytes and TILs. Further, no difference in viability was noted between salt treatment and mannitol treatment in any of the four cohorts mentioned above. Data presented as mean ± standard error of mean (SEM) from 5 independent CD4+T-cell expansions.

To determine the impact of high salt treatment on CD4+T cells, we first studied their proliferation and phenotype switch capabilities under ex vivo cell culture conditions. Studies by Wu et al. [19] demonstrated that treatment of CD4+T cells isolated from splenocytes of wild-type mice when cultured for 3 days at 40 mM excess NaCl induced inflammatory phenotype switch. Similarly, previous dose–response studies reported from our laboratory using cancer cells demonstrated that a treatment with 35–50 mM excess NaCl (final NaCl concentration of 135 to 150 mM) induced inflammatory activation, while concentrations at or above 100 mM excess NaCl were cytotoxic [26,27,31,32]. It is important to note that the basal culture media (e.g., RPMI1640) have 100 mM NaCl, and reducing the NaCl concentration by even 10 mM (final NaCl concentration of 90 mM) is cytotoxic to cells. Therefore, performing low sodium studies is a non-viable option. Therefore, in view of the sustained high salt concentration for long-term treatment used in our current study, we maintained the NaCl treatment conditions at ∆35 mM (or ∆0.035 M NaCl, with a final NaCl concentration of 135 Mm, referred to as “high salt” in this communication). Tumor-primed CD4+T cells were obtained from the draining lymph nodes from tumor bearing mice. Draining lymph nodes (DLNs) and spleens were harvested on day 28 post-injection from tumor bearing C56Bl/6 mice injected in the flank with 0.75 × 10^5^ Py230 syngeneic murine breast tumor cells. The CD4+T cells from a single cell suspension of DLN and spleen were isolated at greater than 95% purity. These isolated cells (initial cell count of 0.3 million CD4+T cells) were expanded ex vivo for five stimulation cycles (Figure 1). Each stimulation cycle lasted for 7 days, consisting of 3 days treatment with anti-CD3/CD28 mAb and ∆35 mM NaCl followed by culture in the presence of an IL-7/IL-23 cocktail along with heat killed Py230 cells for 4 days. An equimolar (35 mM) mannitol was used as the osmotic control in our experiments. Heat killed Py230 cells were added to the cultures to obtain a final CD4+T cell to (heat killed) cancer cell ratio of 1:10. Following five stimulation cycles (day 35), CD4+T cells isolated from DLNs and spleens from tumor bearing mice and spleens from non-tumor bearing mice demonstrated a 10^10^ fold expansion in the presence of high salt, while there was only 10^8^ fold expansion (*p* < 0.05) following equimolar mannitol treatment. This suggested that high salt induced significantly higher cell proliferation. However, there was no significant difference in the cell proliferation based on the site of CD4+T cell procurement (DLNs vs. tumor splenocytes, *p* > 0.8; DLNs vs. non-tumor splenocytes, *p* > 0.8). As shown in Figure 1D, tumor infiltrating lymphocytes (CD4+TILs) demonstrated slower expansion, and there was no difference between high salt treatment and equimolar mannitol treatment. Further, it is important to note that (Figure 1E,F) our lymphocyte culture has consistently resulted in a cell viability of greater than 95% through all five stimulation cycles for CD4+T cells obtained from DLNs, tumor splenocytes, non-tumor splenocytes and TILs. These data suggest that the slower expansion of TILs is not due to cell cytotoxicity but rather the possible terminal differentiation and lack of plasticity of CD4+TIL, which will be further confirmed by CD4 subset analysis. 

**Figure 2:**
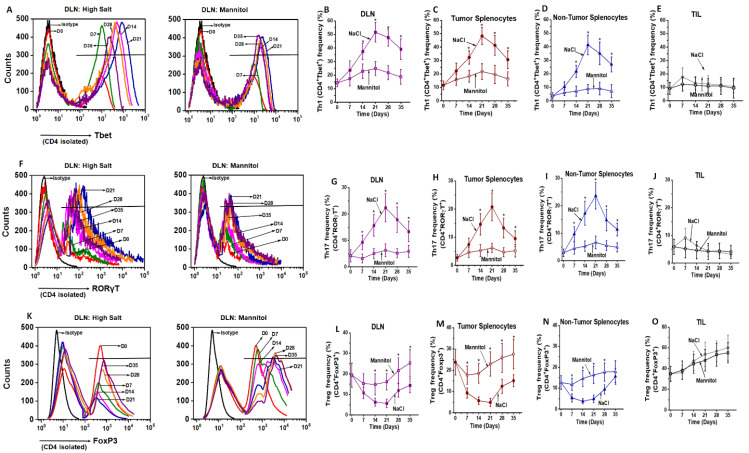
Changes in the effector and suppressive phenotype following ex vivo expansion of CD4+T cells. (**A**–**E**) Flow cytometry analysis of Tbet (Th1) expression of CD4+T cells on day 7, 14, 21, 28 and 35 to study the impact of stimulation cycles (S1–S5, respectively). Isotype is used as negative control and day 0 is used to obtain the baseline phenotype frequencies. CD4+T cells were obtained from DLNs (**A**); Quantitative temporal changes in Tbet (Th1) expression in DLNs (**B**), tumor-splenocytes (**C**), non-tumor splenocytes (**D**) and TILs (**E**) which were subjected to high salt (∆0.035 M NaCl, filled symbols) or equimolar mannitol (0.035 M mannitol, treatment control, open symbols); (**F**–**J**) flow cytometry (**F**) and temporal changes in RORγT (Th17) expression (**G**–**J**) following treatment with high salt and mannitol treatment on CD4+T cells obtained from DLNs (**G**), tumor-splenocytes (**H**), non-tumor splenocytes (**I**) and TILs (**J**); flow cytometry (**K**) and temporal changes (L–O) of FoxP3 (Treg) expression following treatment with high salt and mannitol treatment on CD4+T cells obtained from DLNs (**L**), tumor-splenocytes (**M**), non-tumor splenocytes (**N**) and TILs (**O**). Data obtained from 5 independent measurements and presented as mean ± SEM; (*) *p* < 0.05.

We next studied the phenotype ratios of these extensively proliferated CD4+T cells. As shown in Figure 2A–E, freshly isolated CD4+T cells from DLNs showed a 14.3 ± 2.9% Th1 frequency as studied by Tbet expression. Following high salt treatment, by the end of the third stimulation (S3) there was an increase in Th1 frequency to 51.6 ± 7.2% (*p* < 0.05), while equimolar mannitol control cultures demonstrated a slightly higher 24.9 ± 5.1% (*p* < 0.05) Th1 frequency. Splenocytes from tumor bearing mice under similar treatment conditions also showed an increase from 11.6 ± 3.2% to 48.2 ± 6.4% (*p* < 0.05) under high salt conditions versus 21.8 ± 5.9% (*p* < 0.05) under mannitol treatment conditions by the end of the third stimulation. Similarly, splenocytes from non-tumor bearing mice under similar treatment conditions also showed an increase from 4.7 ± 2.4% to 41.4 ± 7.2% (*p* < 0.05) under high salt conditions versus 8.9 ± 3.4% (*p* = n/s) under mannitol treatment conditions by the end of the third stimulation. Our studies demonstrated that there was a decrease in the Th1 frequency with the fourth and fifth stimulation, as compared to the end of the third stimulation cycle. However, similar high salt treatment on tumor infiltrating lymphocytes (TILs) did not demonstrate any significant increase in Tbet/Th1 expression following high salt treatment, which was from 9.2 ± 4.5% to 12.6 ± 6.3% (*p* = n/s) versus 10.7 ± 5.4% (*p* = n/s) under equimolar mannitol treatment. 

To check for the frequency of Th17 effector phenotype, we measured the expression of RORγT transcription factor in CD4+T cells. By end of three stimulation cycles (Figure 2F–J), high salt treatment enhanced the Th17 frequency from 4.2 ± 2.3% (S0) to 22.4 ± 5.2% (S3), while the equimolar mannitol control did not induce a significantly higher Th17 differentiation (6.3 ± 2.4%, *p* > 0.05). Splenocytes from tumor bearing mice under similar treatment conditions also showed an increase from 2.7 ± 1.2% to 20.8 ± 4.9% (*p* < 0.05, high salt), while it was just a marginal increase 6.7 ± 3.7% (*p* > 0.05) under mannitol treatment conditions. Similarly, splenocytes from non-tumor bearing mice under similar treatment conditions also showed an increase from 2.9 ± 1.4% to 23.6 ± 4.8% (*p* < 0.05) under high salt conditions versus 5.9 ± 2.3% (*p* > 0.05) under mannitol treatment conditions by the end of the third stimulation. The CD4+T cells from TILs under similar treatment conditions did not demonstrate any significant change in RORγT expression, which was from 5.9 ± 2.7% to 4.4 ± 1.9% (*p* = n/s) under high salt conditions versus 4.1 ± 1.8% (*p* = n/s) under mannitol treatment conditions by the end of the third stimulation. To determine the role of high salt on immunosuppressive regulatory CD4+Tcell (Treg) differentiation, we studied the expression of FoxP3. As shown in Figure 2K–O, high salt treatment decreased the Treg frequency from 19.4 ± 5.8% (S0) to 5.7 ± 2.1% (S3), while the equimolar mannitol control did not induce a significant change in Treg frequency (16.1 ± 3.9%, *p* > 0.05). Splenocytes from tumor bearing mice have also shown decreased Treg frequency from 23.8 ± 6.1% to 4.9 ± 1.9% (*p* < 0.05) following high salt conditions, while equimolar mannitol did not induce any significant change (22.9 ± 5.7%, *p* > 0.05). Similarly, splenocytes from non-tumor bearing mice under similar treatment conditions also showed a decrease from 12.7 ± 3.3% to 4.9 ± 1.8% (*p* < 0.05) under high salt conditions versus 16.3 ± 4.1% (*p* > 0.05) under mannitol treatment conditions by the end of the third stimulation. However, high salt treatment on CD4+TILs, by the end of the third stimulation, did not show any difference in the Treg frequency (53.8 ± 11.7%) as compared to equimolar mannitol treatment (47.9 ± 10.3%, *p* = n/s). Taken together, these data suggest that salt mediated an enhanced effector Th1 and Th17 phenotypic switch in CD4+T cells. Further, high salt treatment did not have any significant impact on TILs towards inflammatory activation. This could be because CD4+TILs were already in a hostile immunosuppressive tumor microenvironment and attained terminal differentiation, limiting their ex vivo plasticity.

**Figure 3:**
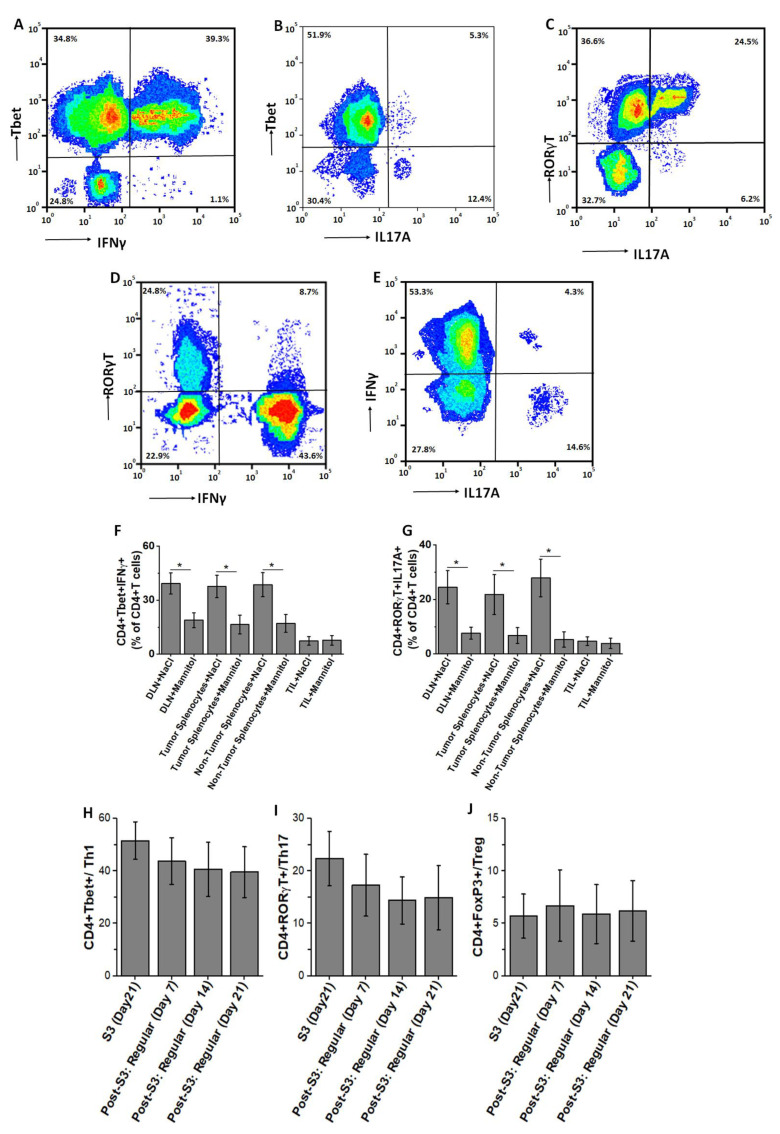
Intracellular cytokine expression in ex vivo expanded CD4+T cells at the end of third high salt stimulation. The ex vivo expanded CD4+T cells were stained for Tbet, IFNγ, RORγT and IL-17A. (**A**–**E**) CD4+T cells obtained from DLNs were stained at the end of third stimulation and following plots were reported: (**A**) Cells stained for Tbet and IFNγ; (**B**) Cells stained for Tbet and IL-17A; (**C**) Cells stained for RORγT and IL-17A; (**D**) Cells stained for RORγT and IFNγ; (**E**) Cells stained for IFNγ and IL-17A. (**F**) Changes in the CD4+Tbet+ IFNγ+ cells at the end of third stimulation cycle among the cells obtained from DLNs, tumor-splenocytes, non-tumor splenocytes, and TILs. (**G**) Changes in the CD4+RORγT+IL17A+ cells at the end of third stimulation cycle among the cells obtained from DLNs, tumor-splenocytes, non-tumor splenocytes, and TILs. (**H**–**J**) Changes in the Th1 (**H**), Th17 (**I**) and Treg (**J**) frequency in the DLN-obtained CD4+T cells at the end of third stimulation cycle under high salt conditions followed by cell culture in the regular basal media for 21 days. Data obtained from 5 independent measurements and presented as mean ± SEM; (*) *p* < 0.05.

We next determined if the inflammatory transcription factor expression correlated with the intracellular inflammatory cytokine profile of these ex vivo differentiated CD4+T cells. As shown in Figure 3A–G, intracellular staining of CD4+T cells at the end of the S3-treatment cycle with high salt obtained from DLNs demonstrated that up to 53% of Tbet+ CD4+T cells expressed Th1 inflammatory cytokine IFNγ. Similarly, 40% of RORγT+CD4+T cells expressed Th17-specific inflammatory cytokine IL-17. Interestingly, only 4.3% of CD4+T cells were double positive for IFNγ and IL-17. These data suggest that high salt-mediated enhanced expression of Th1 and Th17 specific transcription factors, Tbet and RORγT, respectively, correlated with the expression of subset specific cytokines, namely IFNγ and IL-17, respectively. Further, we verified if the high salt-mediated phenotype switch of CD4+T cells was retained following reversal to regular (non-high salt) culture conditions. As shown in Figure 3H–J, following the end of the third stimulation with high salt, these activated CD4+T cells, when cultured in regular basal conditions, retained their phenotype switch. These data suggest that ex vivo high salt treatment induced a potent effector activation which was maintained even after switch to regular culture conditions. 

**Figure 4:**
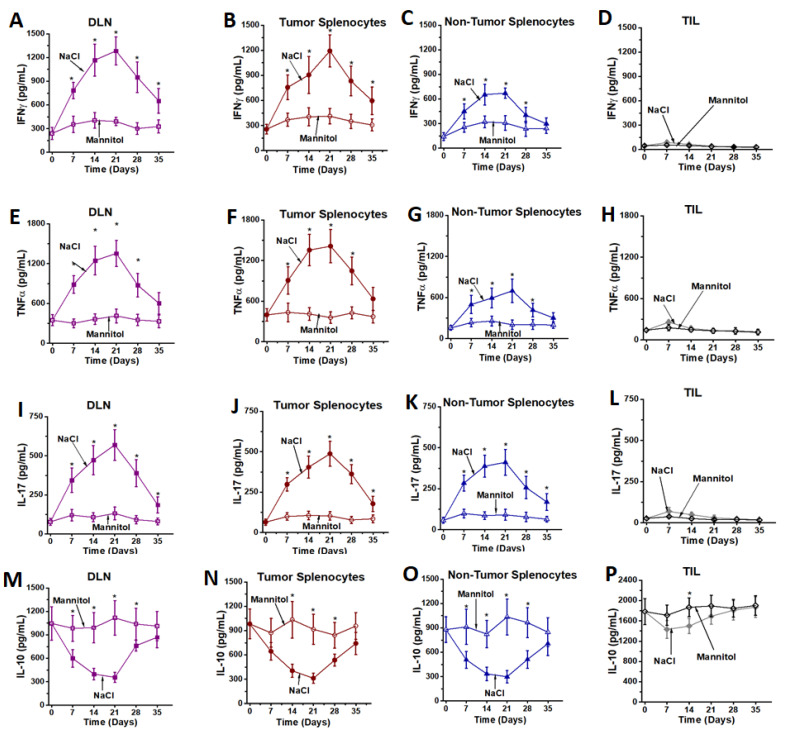
Cytokine expression from ex vivo expanded CD4+T cells. Inflammatory cytokines—IFNγ (**A**–**D**), TNFα (**E**–**H**) and IL-17 (**I**–**L**); and immunosuppressive cytokine, IL-10 (**M**–**P**) were analyzed in CD4+T cells obtained from DLNs (**A**,**E**,**I**,**M**), tumor splenocytes (**B**,**F**,**J**,**N**), non-tumor splenocytes (**C**,**G**,**K**,**O**) and TILs (**D**,**H**,**L**,**P**) following treatment with high salt (filled symbols) and equimolar mannitol (open symbols). Data obtained from 5 independent measurements and presented as mean ± SEM; (*) *p* < 0.05.

To determine if the intracellular cytokine expression correlated with the secretion of inflammatory cytokines, we tested the secretory ability of cytokines from these high salt activated CD4+T cells by ELISA. As shown in Figure 4, high salt treatment enhanced the secretion of inflammatory cytokines (IFNγ, TNFα and IL-17) from DLNs, tumor splenocytes and non-tumor splenocytes, as compared to respective equimolar mannitol treatment. Conversely, high salt treatment decreased the expression of anti-inflammatory IL-10 cytokine from DLNs, tumor splenocytes and non-tumor splenocytes, as compared to respective equimolar mannitol treatment. However, high salt had no significant impact of the pro- or anti-inflammatory cytokines on CD4+T cells from TILs. These data suggest that high salt induced effector pro-inflammatory differentiation of tumor-primed and non-tumor primed CD4+T cells, while the TILs, due to lack of plasticity and exposure to a hostile tumor microenvironment, could not undergo inflammatory differentiation.

### 3.2. High Salt Activated CD4+T Cells Induce Tumor Regression

**Figure 5:**
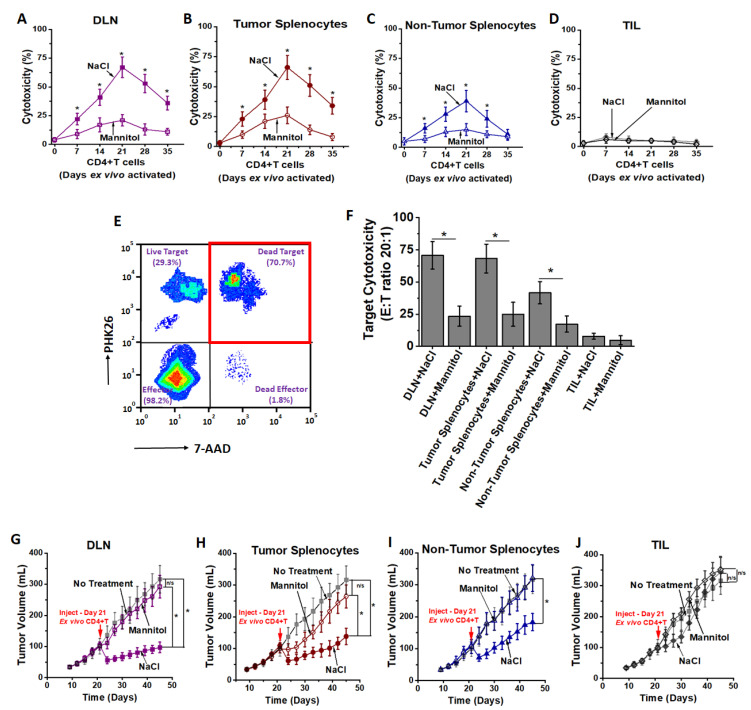
Anti-cancer efficacy of ex vivo expanded CD4+T cells. (**A**–**C**) Cytotoxic efficiency of CD4+T cells obtained from DLNs (**A**), tumor splenocytes (**B**), non-tumor splenocytes (**C**) and TILs (**D**) following high salt (filled symbols) and equimolar mannitol (open symbols) treatment for five stimulation cycles (up to day 35) tested on Py230 breast cancer cells at effector-to-target (E:T) ratio of 20:1 analyzed by LDH release assay. It is to be noted that DLNs, tumor splenocytes and TILs were obtained from tumor-bearing mice day 28 post-injection of Py230 breast cancer cells into C57Bl/6 mice. (**E**,**F**) Cytotoxicity of target cells was confirmed by staining of target cells with PKH26 and double positive events (PHK26 and 7-AAD) were determined. (**E**) Flow cytometry plot with CD4+T cells obtained from DLNs at the end of third stimulation cycle on Py230 breast cancer cells at E:T ratio of 20:1. (**F**) Changes in the cytotoxic efficiency of CD4+T cells at the end of third stimulation cycle obtained from DLNs, tumor-splenocytes, non-tumor splenocytes and TILs. Data obtained from 5 independent measurements and presented as mean ± SEM; (*) *p* < 0.05. (**G**–**J**) In vivo anti-tumor efficacy of ex vivo expanded CD4+T cells obtained from DLNs (**G**), tumor splenocytes (**H**), non-tumor splenocytes (**I**) and TILs (**J**) following high salt (filled symbols) and equimolar mannitol (open symbols); grey line and symbol represent Py230 tumors in C57Bl/6 mice without any treatment. Data represented as mean ± SEM with *n* = 6 mice in each cohort, *p* < 0.05.

As high salt induced differentiation to effector CD4+T cell phenotype, we next determined the anti-cancer efficiency of these activated CD4+T cells. As shown in Figure 5A–D, the DLN derived CD4+T cells from day 21 post-stimulation (S3) demonstrated 67 ± 9% cytotoxicity as analyzed by LDH release assay (E:T ratio 20:1), as compared to 21 ± 5% (*p* < 0.05) cytotoxicity by equimolar mannitol stimulated CD4+T cells under similar cell culture conditions. The CD4+T cells (S3) obtained from splenocytes from tumor-bearing mice following high salt treatment demonstrated a 65 ± 11% cytotoxicity, compared to 26 ± 7% (*p* < 0.05) cytotoxicity with equimolar mannitol activation. However, splenocytes obtained from non-tumor bearing mice following high salt treatment demonstrated a significantly lower cytotoxicity (39 ± 9%, *p* < 0.05 for both DLN and tumor-splenocyte comparator cohorts). Importantly, CD4+T cells obtained from TILs did not demonstrate significant cytotoxicity following high salt treatment (6 ± 2%,) as compared to equimolar mannitol treatment (5 ± 3%, *p* = n/s). To further validate our LDH release assay data, and to determine if the cytotoxicity resulted from target cell (cancer cell) death and not effector cell (CD4+T cell) death, we labeled the cancer cells with PHK26 and performed flow cytometry-based cytotoxicity studies. As shown in Figure 5E,F, up to 98% of the observed cell death is from target cells (cancer cells). These data demonstrated that CD4+T cells from DLNs and splenocytes from tumor-bearing mice were primed for tumor-recognition and were hence able to mount an efficient anti-cancer response. 

To further determine the in vivo anti-tumor efficiency of ex vivo activated CD4+T cells, we have utilized a Py230 breast cancer cell based orthotopic syngeneic murine model. The CD4+T cells following three ex vivo stimulations were intraperitoneally injected into 21 day old tumor bearing mice and followed up until day 45. As shown in Figure 5G–J, high salt treated CD4+T cells obtained from all three sources (DLNs, tumor-splenocytes and non-tumor splenocytes) demonstrated significant anti-tumor efficiency compared with the respective mannitol treated CD4+T cells. Among the high salt activated CD4+T cell treated cohorts, CD4+T cells obtained from DLNs demonstrated significantly reduced tumor growth (day 45, high salt: 97 ± 24 mm^3^ vs. mannitol: 292 ± 46 mm^3^, *p* < 0.05). These in vivo data along with the in vitro cytotoxicity studies demonstrated that tumor-primed CD4+T cells from DLNs exerted the highest anti-cancer response.

### 3.3. High Salt Activated CD4+T Cells Utilize Glycolytic Metabolic Pathway

**Figure 6:**
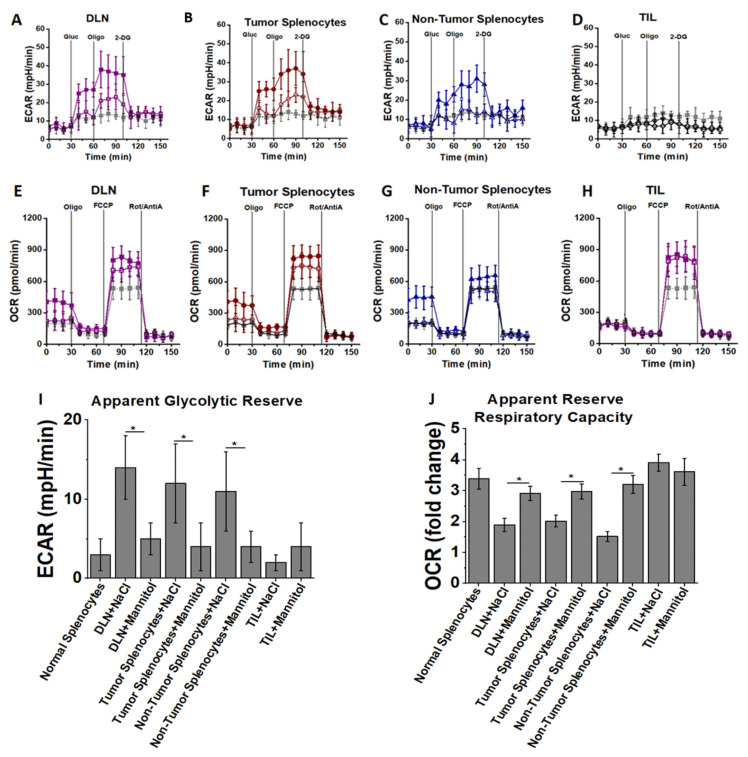
High salt induces metabolism with unique oxidative fuel capacity. CD4+T cells obtained from DLNs (**A**,**E**), tumor splenocytes (**B**,**F**), non-tumor splenocytes (**C**,**G**) and TILs (**D**,**H**) at the end of third stimulation cycle (day 21) were cultured in base DMEM media without glucose and glutamine. Extracellular acidification rate (ECAR) (**A**–**D**) was assessed by time-course changes in lactate production after the addition of 25 mM glucose (gluc) and in response to the metabolic inhibitors oligomycin (oligo) and 2DG. For oxygen consumption rate (OCR) (**E**–**H**), cells at the same stimulation cycle mentioned above were cultured in base DMEM media with 25 mM glucose for basal OCR response, followed by time-course response changes with addition of mitochondrial inhibitors oligomycin, FCCP, and rotenone and antimycin A (Rot/AntiA). Apparent glycolytic reserve (**I**) calculated from ECAR analysis and apparent reserve respiratory capacity (**J**) calculated from OCR are shown. Data obtained from 5 independent measurements and presented as mean ± SEM; (*) *p* < 0.05.

The above mentioned experimental evidence strongly suggested that high salt treatment induces effector activation of CD4+T cells, and we next determined the metabolic changes in the CD4+T cells mediated by high salt treatment. Studies from other laboratories have shown that the effector phenotypic switch of CD4+T cells is associated with higher glycolytic oxidation rates, while Tregs are associated with high mitochondrial oxidation. As effector phenotypes of CD4+T cells express high quantities of inflammatory proteins and thus have predominant anabolic metabolism (over catabolic metabolism), glycolytic oxidation will produce the required metabolites for anabolic macromolecule building cellular processes. To determine the high salt induced metabolic changes following three stimulation cycles (day 21) noted above, the lactate production and oxygen consumption were measured using an extracellular metabolic flux analyzer. For measuring the extracellular acidification rate (ECAR), cells were cultured in the absence of glucose, and lactate production was measured following sequential addition of: 25 mM glucose; oligomycin, to block mitochondrial ATP production and promote maximal rates of glycolysis; and finally 2-DG to inhibit glycolysis. As shown in Figure 6A–D, high salt treatment increased ECAR following glucose addition, along with a robust increase in ECAR following treatment with oligomycin, indicating that high salt treatment could enhance lactate production and therefore have higher glycolytic reserve. Taken together, these data indicate that high salt activated CD4+T cells, as compared to mannitol treatment, have an increased glycolytic capacity to generate ATP as needed to build pro-growth metabolites required for inflammatory effector pathways.

To determine mitochondrial oxidative metabolism, the oxygen consumption rate (OCR) was measured. As shown in Figure 6E–H, prior to treatment with metabolic inhibitors, high salt treated CD4+T cells demonstrated higher mitochondrial oxygen consumption. Mitochondrial oxidation is important for CD4+T cell activation and proliferation. The suppression of mitochondrial ATP production by oligomycin reduced oxygen consumption in all groups (high salt and mannitol treated) to equivalent levels, suggesting oxygen consumption is directly proportional to mitochondrial ATP production. Following treatment with FCCP, an uncoupler of oxidative phosphorylation with electron transport, to induce maximal mitochondrial respiration, high salt treatment showed slightly higher (*p* = n/s) OCR. However, when compared to basal oxygen consumption, the apparent reserve respiratory capacity was higher in the control group. Thus, high salt treatment induced pro-inflammatory CD4+T cell differentiation which has high glycolytic capacity (Figure 6I) and high basal mitochondrial oxygen consumption (Figure 6J), indicating that high salt treatment promotes CD4+T cell activation and metabolic capacity to enhance anabolic macromolecules needed for inflammatory response.

### 3.4. NFAT5 Mediates High Salt Mediated Effector CD4+T Cell Differentiation

**Figure 7:**
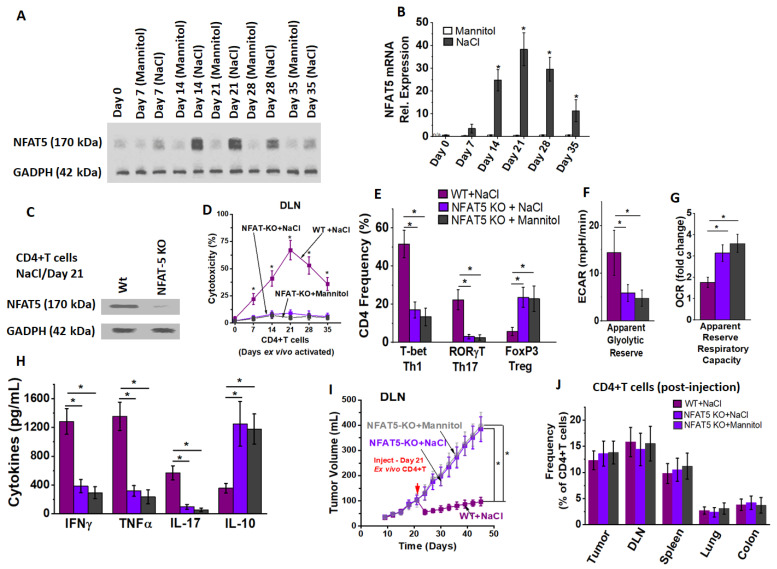
Critical role of NFAT5 in high salt-mediated effector responses. (**A**,**B**) NFAT5 expression analysis in CD4+T cells obtained from DLNs following high salt and equimolar mannitol treatment by Western blot (**A**) and qRT-PCR (**B**). NFAT5 expression analysis in CD4-specific NFAT5 knock out mice. CD4+T cells were obtained from the day 21 DLNs of the tumor bearing CD4+NFAT5-KO mice post-injection of Py230 breast cancer cells followed by three high salt stimulation cycles mentioned above (ex vivo day 21). (**C**) NFAT5 expression at the end of three ex vivo high salt stimulation cycles in day 21 DLN CD4+T cells obtained from wildtype (Wt) and CD4+NFAT5-KO (NFAT-5 KO) mice. (**D**) Cytotoxicity of CD4+T cells obtained from DLNs of CD4+NFAT5-KO tumor model following high salt treatment (purple square), DLNs of CD4+NFAT5-KO tumor model following equimolar mannitol treatment (grey square) and DLNs from wild-type following similar high salt treatment (magenta squares) tested on Py230 breast cancer cells at effector-to-target (E:T) ratio of 20:1. (**E**–**H**) Th-phenotypic frequencies (**E**), apparent glycolytic reserve (**F**), apparent reserve respiratory capacity (**G**) and cytokine expression (**H**) on the ex vivo high salt expanded CD4+T cells obtained from CD4+NFAT5-KO mice (purple square). The mannitol expanded CD4+T cells obtained from CD4+NFAT5-KO mice (grey squares) and high salt expanded CD4+T cells obtained from wild-type (magenta squares) were also shown. All cellular data were obtained from 5 independent measurements and presented as mean ± SEM; (*) *p* < 0.05. (**I**) Changes in tumor volume following injection on day 21 into Py230 orthotopic syngeneic tumors in wild type with CD4+T cells obtained from DLNs of CD4+NFAT5-KO tumor model following high salt treatment (purple square), DLNs of CD4+NFAT5-KO tumor model following equimolar mannitol treatment (grey square) or from wild-type following similar high salt treatment (magenta squares). (**J**) Tumor and organ localization of injected CTV labelled CD4+T cells (ex vivo expanded) obtained from DLNs of CD4+NFAT5-KO tumor model following high salt treatment (purple), DLNs of CD4+NFAT5-KO tumor model following equimolar mannitol treatment (grey) or from wild-type following similar high salt treatment (magenta). Data presented and percentage of CD4+T cells in the tumor, DLNs, spleen lung and colon to the total number of resident CD4+T cells in that respective tumor or organ. Data represented as mean ± SEM with *n* = 6 mice in each cohort, *p* < 0.05.

As the high salt-mediated inflammatory phenotype switch of CD4+T cells suggested a tonicity-mediated change, we tested if the tonicity specific transcription factor TonEBP/NFAT-5, a known transcription factor to induce cellular changes following osmotonic stress [23,33,34,35], played a role in this CD4+T cell differentiation. As shown in Figure 7A,B, there was the highest increase in NFAT5 expression by day 21 following the third high salt stimulation cycle. To determine if TonEBP/NFAT5 directly played a role in the inflammatory phenotypic differentiation of CD4+T cells following high salt ex vivo treatment, we have utilized a murine model with CD4-specific knock out of TonEBP/NFAT5 (CD4^cre/cre^TonEBP^flox/flox^C57Bl/6 murine model, will be referred to as CD4+NFAT5-KO). As expected there was no expression of NFAT5 at the end of three high salt stimulation cycles in the CD4+T cells obtained from day 21 DLNs of tumor bearing CD4+NFAT5-KO mice (Figure 7C). The CD4+T cells obtained from DLNs of tumor bearing CD4+NFAT5-KO mice following three stimulations with high salt did demonstrate a cytotoxicity (Figure 7D) of just 11 ± 4% (wild-type: 67 ± 9%; *p* < 0.05) on py230 breast cancer cells (E:T ratio 20:1). Importantly, there was no significant difference in the cytotoxic ability between high salt treatment and equimolar mannitol treatment (high salt: 11 ± 4% vs. mannitol: 9 ± 5%, *p* = n/s) on DLN CD4+T cells from CD4+NFAT5-KO. Phenotype and cytokine analysis (Figure 7E–H) of these CD4+T cells (S3) from transgenic mice demonstrated a lowered effector (Th1, Th17) phenotype switch and enhanced immunosuppressive (Treg) phenotype following both high salt and equimolar mannitol treatment. Further metabolic analysis demonstrated that NFAT5-KO in CD4+T cells decreased the glycolytic reserve capacity. In vivo intraperitoneal injection of CD4+T cells from DLNs of CD4+NFAT5-KO (following three high salt stimulations) into day 21 orthotopic syngeneic tumors in wild type mice demonstrated a lack of anti-tumor response (Figure 7I). Importantly, we noted no difference (Figure 7J) in the tumor localization of injected CD4+T cells (and also localization to other secondary lymphoid organs and solid organs) between lymphocytes obtained from wild-type versus NFAT5-KO mice. This suggested that the anti-cancer impact of high salt activated wild-type CD4+T cells is not due to enhanced tumor localization. Taken together, these data strongly indicate that NFAT5 plays a critical role in the inflammatory effector phenotypic differentiation of CD4+T cells following high salt activation.

**Figure 8:**
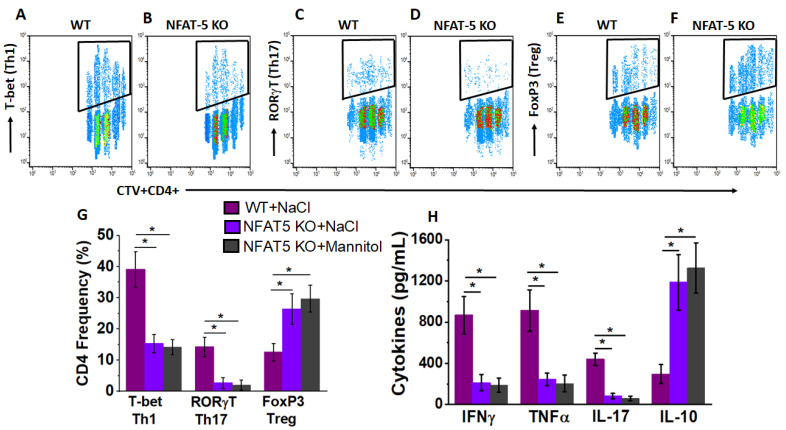
In vivo tracking and localization of ex vivo expanded CD4+T cells. Analysis of Tbet (**A**,**B**), RORγT (**C**,**D**) and FoxP3 (**E**,**F**) in CTV stained CD4+T cells injected on day of tumor-bearing wild-type mice. High salt expanded CD4+T cells for three stimulation cycles obtained from DLNs of wild type (**A**,**C**,**E**) and CD4+NFAT5-KO (**B**,**D**,**F**) were analyzed for tumor localization. (**G**,**H**) Th-phenotypic frequencies (**G**) and cytokine expression (**H**) of the tumor localized ex vivo expanded CD4+T cells obtained from high salt treated wild-type mice (magenta), high salt treated (purple), and equimolar mannitol treated CD4+NFAT5-KO mice (grey). Data obtained from *n* = 3 in each cohort and presented as mean ± SEM; (*) *p* < 0.05.

To determine the tumor localization of injected CD4+T cells (following high salt stimulation), we performed in vivo cell tracking. As shown in Figure 8, the day 45 explanted tumors had a 39.1 ± 5.7% localization of Th1 phenotype and 14.3 ± 3.1% localization of Th17 phenotype from ex vivo activated CD4+T cells. Further, our data suggested the CD4+T cells have undergone approximately five cell divisions, indicating that the ex vivo injected CD4+T cells were able to exert a viable robust anti-tumor response. As expected, NFAT5-KO in CD4+T cells had significantly lower tumor localization with effector phenotype. Taken together, our data indicate that ex vivo activation of tumor-primed CD4+T cells with high salt resulted in a strong anti-cancer response with a strong futuristic possibility to adopt similar human cell-based immunotherapeutic approaches. 

## 4. Discussion 

**Figure 9:**
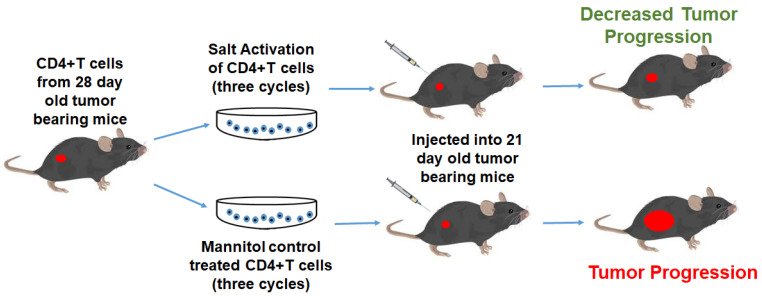
Schematic of the ex vivo high salt expansion of CD4+T cells.

Several adoptive T cell-based immunotherapeutic approaches have recently emerged in cancer treatment strategies [36]. The central purpose of all these strategies is to stimulate a potent, viable, tumor-specific, T-cell response with a retained in vivo expansion capability [37]. While therapeutic approaches using engineered T-cells with chimeric tumor associated antigens (CAR-T cells) are growing in popularity [38], several other bulk expansion strategies such as using tumor infiltrating lymphocytes (TILs) ex vivo expanded with IL-2 [39], or lymphodepletion [40] followed by autologous transfer of activated T cells were also extensively tested in clinical trials. A major challenge with the bulk expansion strategies includes the lack of tumor antigen specificity [41]. Further, CAR-T cells are developed with generic specificity to cancer neo-antigens (off-the-shelf), but this approach would be difficult to generate specific T-cells for personalized use [42]. For bulk expansion, cytokines such as IL-2 have been shown to expand the immunosuppressive Treg phenotype of T-cells, thus limiting the long-term success of this strategy [43]. In this communication, we utilized a novel approach to expand the effector phenotype of CD4+T cells which retained their in vivo anti-cancer efficiency (Figure 9). Conceptually, salt is a well-known inflammatory agent with a potential to activate Th17/CD4+T cell responses in autoimmune EAE [19], graft-versus host disease (GvHD) [20], gut commensal expansion of Th17 [44], and heart ailment-based preclinical models [45]. Interestingly, Willebrand et al. have reported that a high salt diet in murine tumor models has shown enhanced inflammatory T-cell activation, along with the depletion of anti-inflammatory and pro-tumor myeloid derived suppressor cells [46]. In line with this evidence, He et al. have recently shown that a high salt diet induced anti-cancer effect through NFAT5-mediated inflammatory activation of immune cells [47]. While in vivo activation by a high salt diet could theoretically activate CD4+T cells in cancer patients, a potential recommendation of a high salt diet as a practical application is impossible. 

To specifically induce the expansion of the effector phenotype of CD4+T cells, we have utilized NaCl (salt), a known inflammatory molecule, for ex vivo treatment. As noted above, a major challenge with ex vivo expansion is tumor-specificity. We have observed that CD4+T cells obtained from DLNs and splenocytes demonstrated the highest anti-cancer response. However, CD4+T cells from non-tumor-primed spleens produced a diminished anti-cancer cell cytotoxicity. To maintain the tumor specificity, we have added heat killed syngeneic cancer cells in our culture during ex vivo expansion. Previous studies by Gerriets et al. [30,48] have demonstrated that the effector phenotypic switch of CD4+T cells is associated with a higher rate of glycolytic activity with relatively higher levels of basal mitochondrial activity. Our current studies are in agreement with these findings, in that tumor-primed CD4+T cells following high salt activation demonstrated a three times higher level of glycolytic reserve and 1.8 times higher basal mitochondrial oxidation, as compared to equimolar mannitol control stimulation. However, the apparent reserve mitochondrial respiratory capacity was higher in mannitol control treated CD4+T cells, thus suggesting that high salt treatment makes CD4+T cells utilize a higher basal mitochondrial oxidation. These data suggest that these specific changes in glycolytic and mitochondrial metabolic functionality following high salt treatment allow CD4+T cells to produce anabolic macromolecules such as inflammatory cytokines and cytotoxic granular needed for anti-cancer effector activity. Studies from various laboratories have confirmed the upregulation of AMPK and mTOR mediated metabolic signaling pathways during the effector activation of CD4+T cells [49,50,51]. Further, our studies showed that excessive stimulation (fourth and fifth cycle) with high salt caused immune-exhaustion, suggesting that uncontrolled prolonged high salt stimulation on clinical samples might not result in effective anti-cancer response. Future studies to specifically probe the signaling pathways and transcription factor changes will be helpful to further delineate the molecular mechanism. 

A high salt associated tonicity stress mediating upregulation of the transcription factor NFAT5 (nuclear factor of activated T cells, also known as tonicity-responsive enhancer-binding protein, TonEBP) resulting in inflammatory responses is well-documented in cardiac and renal diseases [52,53,54]. NFAT5 is known to play a critical role in the high salt-mediated exacerbation of autoimmune diseases along with the induction of inflammatory Th17 CD4+T cell phenotype [53]. Studies by Kleinewietfeld et al. demonstrated that a high salt diet induced NFAT5 dependent Th17 polarization and upregulation of the pro-inflammatory cytokines IL-2, granulocyte monocyte-colony stimulating factor (GM-CSF) and TNF-α through the P38/MAPKinase signaling pathway [53]. Tellechea et al., using lung and ovarian cancer models, have shown that NFAT5 overexpression in macrophages induced an IFNγ/Th1 polarization of CD4+T cells, while NFAT5 knock down in macrophages showed a reduced tumor infiltration of cytotoxic CD8+T cells to the tumor site [55]. Interestingly, studies by Alberdi et al. demonstrated that NFAT5 exerted a context-specific activation of CD4+T cells depending upon ex vivo versus in vivo hyperosmotic activating conditions, with NFAT5 exerting a higher inflammatory phenotype switch in CD4+T cells when activated under ex vivo conditions [52]. Further, multiple research groups have demonstrated that NFAT5 knock down in CD4+T cells abolished the expression of Th17-specific transcription factor RORγT and Th17-specific cytokine, IL-17 [56]. In line with these findings, we have shown that CD4-specific knock out of NFAT5 abrogated an effector phenotypic switch of tumor-primed CD4+T cells, leading to lowered anti-cancer cytotoxicity. These data point out the critical role of NFAT5 upregulation in high salt-mediated CD4+T cell based immunotherapy. In the future, it would be interesting to test if direct engineering of the NFAT5 gene into T-cells will further enhance the functionality of other adoptive T cell therapies such as CAR-T cells. A nagging problem with adoptive T cell transfer approaches is the viability and tumor localization of adoptively transferred immune cells. Our studies have demonstrated that ex vivo high salt expanded CD4+T cells retained cell viability and have undergone five cell cycle divisions. Thus, supporting the notion that our approach resulted in viable effector phenotype to exert a long-term anti-tumor response. It would be very important to test the potential translatable capability of our current approach in a human clinical setting. However, it is important to note that just like the concern with CAR-T cells, there is a challenge with the tumor localization of the injected ex vivo activated CD4+T cells. Theoretically, obtaining a 50–70% tumor localization would have been ideal, but more molecular and cell modification strategies would be needed to attain this higher level of tumor localization. In our hands, we found that only 12–15% of tumor infiltrating lymphocytes were from ex vivo activated CD4+T cells. Novel strategies, such as the transfection of tumor migratory chemokines and their receptors, CXCL11 [57] and CCR2 [58], have been utilized to enhance the tumor localization of CAR-T cells with significant success. A similar approach could be utilized in our ex vivo expanded CD4+T cells. 

An important side-effect of adoptive transfer-based cancer therapeutic approaches is the cytokine storm phenomenon [59], which results from excessive release of inflammatory molecules by ex vivo activated immune cells. Our current studies have not directly addressed this side-effect; however, within the 45 day follow up of our murine breast tumor models, we have not noticed enhanced mortality, suggesting a life-threatening cytokine storm release might not have occurred under the experimental conditions followed in this report. However, future studies with a detailed analysis of peripheral blood and post-mortem organs for inflammatory damage are warranted to determine the absence of the cytokine storm in our ex vivo expansion approach. It would be of importance to perform future studies to determine the role of the infiltration of these ex vivo activated CD4+T cells on other solid organs. Our preclinical model demonstrated that CD4+T cells from DLNs exerted higher anti-tumor efficiency following high salt treatment. It would be more interesting if high salt treatment on circulating CD4+T lymphocytes of human cancer patients could be similarly activated to exert anti-cancer effect. A human-based study with peripheral circulating CD4+T cells adopting the protocol reported in this communication will provide better clinical translational impact of our current study. Additionally, in our current study we did not directly address the role of naïve CD4+T cell (CD45RA) activation versus memory CD4+T cell (CD45RO) expansion following high salt treatment. Future studies will be targeted at delineating the impact of high salt on central memory T cell expansion. Further, although our current studies were limited to a preclinical breast cancer model, we could easily envision the application of this strategy in other solid organ and hematological cancers, which would require further testing in other cancer models.

In conclusion, we report a novel ex vivo strategy for the expansion of CD4+T cells with efficient effector activation using high salt-based culture conditions. Importantly, our approach has enabled the retention of tumor-specificity along with producing effector CD4+T cells which retained long-term in vitro viability. This could have a strong potential for futuristic clinical application towards a combinatorial approach in cancer therapy.

## Data Availability

No archived data anywhere.

## References

[1] Waldman A.D., Fritz J.M., Lenardo M.J. (2020). A guide to cancer immunotherapy: From T cell basic science to clinical practice. Nat. Rev. Immunol..

[2] Thompson E.D., Enriquez H.L., Fu Y.-X., Engelhard V.H. (2010). Tumor masses support naive T cell infiltration, activation, and differentiation into effectors. J. Exp. Med..

[3] Xia A., Zhang Y., Xu J., Yin T., Lu X.-J. (2019). T cell dysfunction in cancer immunity and immunotherapy. Front. Immunol..

[4] Manfredi F., Cianciotti B.C., Potenza A., Tassi E., Noviello M., Biondi A., Ciceri F., Bonini C., Ruggiero E. (2020). TCR redirected T cells for cancer treatment: Achievements, hurdles, and goals. Front. Immunol..

[5] Rapoport A.P., Stadtmauer E.A., Aqui N., Vogl D.T., Chew A., Fang H.-B., Janofsky S., Yager K., Veloso E., Zheng Z. (2009). Rapid immune recovery and graft-versus-host disease–like engraftment syndrome following adoptive transfer of costimulated autologous T cells. Clin. Cancer Res..

[6] Dudley M.E., Wunderlich J.R., Robbins P.F., Yang J.C., Hwu P., Schwartzentruber D.J., Topalian S.L., Sherry R., Restifo N.P., Hubicki A.M. (2002). Cancer regression and autoimmunity in patients after clonal repopulation with antitumor lymphocytes. Science.

[7] Laport G.G., Levine B.L., Stadtmauer E.A., Schuster S.J., Luger S.M., Grupp S., Bunin N., Strobl F.J., Cotte J., Zheng Z. (2003). Adoptive transfer of costimulated T cells induces lymphocytosis in patients with re-lapsed/refractory non-Hodgkin lymphoma following CD34+-selected hematopoietic cell transplantation. Blood.

[8] Hay K.A., Hanafi L.-A., Li D., Gust J., Liles W.C., Wurfel M.M., López J.A., Chen J., Chung D., Harju-Baker S. (2017). Kinetics and biomarkers of severe cytokine release syndrome after CD19 chimeric antigen receptor–modified T-cell therapy. Blood.

[9] Sim G.C., Martin-Orozco N., Jin L., Yang Y., Wu S., Washington E., Sanders D., Lacey C., Wang Y., Vence L. (2014). IL-2 therapy promotes suppressive ICOS+ Treg expansion in melanoma patients. J. Clin. Investig..

[10] Pandiyan P., Zheng L., Ishihara S., Reed J., Lenardo M.J. (2007). CD4+CD25+Foxp3+ regulatory T cells induce cytokine deprivation–mediated apoptosis of effector CD4+ T cells. Nat. Immunol..

[11] Chinen T., Kannan A.K., Levine A.G., Fan X., Klein U., Zheng Y., Gasteiger G., Feng Y., Fontenot J.D., Rudensky A.Y. (2016). An essential role for the IL-2 receptor in Treg cell function. Nat. Immunol..

[12] Sakaguchi S., Yamaguchi T., Nomura T., Ono M. (2008). Regulatory T cells and immune tolerance. Cell.

[13] Robinson T.O., Schluns K.S. (2017). The potential and promise of IL-15 in immuno-oncogenic therapies. Immunol. Lett..

[14] Jeong G.H., Lee K.H., Lee I.R., Oh J.H., Kim D.W., Shin J.W., Kronbichler A., Eisenhut M., Van Der Vliet H.J., Abdel-Rahman O. (2019). Incidence of capillary leak syndrome as an adverse effect of drugs in cancer patients: A systematic review and meta-analysis. J. Clin. Med..

[15] Dutcher J.P., Schwartzentruber D.J., Kaufman H.L., Agarwala S.S., Tarhini A.A., Lowder J.N., Atkins M.B. (2014). High dose interleukin-2 (Aldesleukin)—Expert consensus on best management practices-2014. J. Immunother. Cancer.

[16] Andersen R., Westergaard M.C.W., Kjeldsen J.W., Muller A., Pedersen N.W., Hadrup S.R., Met O., Seliger B., Kromann-Andersen B., Hasselager T. (2018). T-cell responses in the microenvironment of primary renal cell carcinoma-implications for adoptive cell therapy. Cancer Immunol. Res..

[17] Rohaan M.W., Van den Berg J.H., Kvistborg P., Haanen J. (2018). Adoptive transfer of tumor-infiltrating lymphocytes in melanoma: A viable treatment option. J. Immunother. Cancer.

[18] Dar H.Y., Singh A., Shukla P., Anupam R., Mondal R.K., Mishra P.K., Srivastava R.K. (2018). High dietary salt intake correlates with modulated Th17-Treg cell balance resulting in enhanced bone loss and im-paired bone-microarchitecture in male mice. Sci. Rep..

[19] Wu C., Yosef N., Thalhamer T., Zhu C., Xiao S., Kishi Y., Regev A., Kuchroo V.K. (2013). Induction of pathogenic TH17 cells by inducible salt-sensing kinase SGK1. Nature.

[20] Hernandez A.L., Kitz A., Wu C., Lowther D.E., Rodriguez D.M., Vudattu N., Deng S., Herold K.C., Kuchroo V.K., Kleinewietfeld M. (2015). Sodium chloride inhibits the suppressive function of FOXP3+ regulatory T cells. J. Clin. Investig..

[21] Aguiar S.L.F., Miranda M.C.G., Guimaraes M.A.F., Santiago H.C., Queiroz C.P., Cunha P.D.S., Cara D.C., Foureaux G., Ferreira A.J., Cardoso V.N. (2017). High-salt diet induces IL-17-dependent gut inflammation and exacerbates colitis in mice. Front. Immunol..

[22] Lee E., Kim N., Kang J., Yoon S., Lee H.A., Jung H., Kim S.H., Kim I. (2020). Activated pathogenic Th17 lymphocytes induce hypertension following high-fructose intake in Dahl salt-sensitive but not Dahl salt-resistant rats. Dis. Models Mech..

[23] Lee N., Kim D., Kim W.-U. (2019). Role of NFAT5 in the immune system and pathogenesis of autoimmune diseases. Front. Immunol..

[24] Babaer D., Amara S., Ivy M., Zhao Y., Lammers P.E., Titze J.M., Tiriveedhi V. (2018). High salt induces P-glycoprotein mediated treatment resistance in breast cancer cells through store operated calcium influx. Oncotarget.

[25] Faustino-Rocha A., Oliveira P.A., Pinho-Oliveira J., Teixeira-Guedes C., Soares-Maia R., da Costa R.G., Colaco B., Pires M.J., Colaco J., Ferreira R. (2013). Estimation of rat mammary tumor volume using caliper and ultrasonography measurements. Lab. Anim..

[26] Amara S., Majors C., Roy B., Hill S., Rose K.L., Myles E.L., Tiriveedhi V. (2017). Critical role of SIK3 in me-diating high salt and IL-17 synergy leading to breast cancer cell proliferation. PLoS ONE.

[27] Amara S., Ivy M.T., Myles E.L., Tiriveedhi V. (2016). Sodium channel gammaENaC mediates IL-17 synergized high salt induced inflammatory stress in breast cancer cells. Cell Immunol..

[28] Babaer D., Amara S., McAdory B.S., Johnson O., Myles E.L., Zent R., Rathmell J.C., Tiriveedhi V. (2019). Oli-godeoxynucleotides ODN 2006 and M362 exert potent adjuvant effect through TLR-9/-6 synergy to exaggerate mammaglobin-A peptide specific cytotoxic CD8+T lymphocyte responses against breast cancer cells. Cancers.

[29] Babaer D., Zheng M., Ivy M.T., Zent R., Tiriveedhi V. (2019). Methylselenol producing selenocompounds enhance the efficiency of mammaglobin-A peptide vaccination against breast cancer cells. Oncol. Lett..

[30] Gerriets V.A., Kishton R.J., Nichols A.G., Macintyre A.N., Inoue M., Ilkayeva O., Winter P.S., Liu X., Priyadharshini B., Slawinska M.E. (2015). Metabolic programming and PDHK1 control CD4+ T cell subsets and inflammation. J. Clin. Investig..

[31] Amara S., Alotaibi D., Tiriveedhi V. (2016). NFAT5/STAT3 interaction mediates synergism of high salt with IL-17 towards induction of VEGF-A expression in breast cancer cells. Oncol. Lett..

[32] Amara S., Zheng M., Tiriveedhi V. (2016). Oleanolic acid inhibits high salt-induced exaggeration of war-burg-like metabolism in breast cancer cells. Cell Biochem. Biophys..

[33] Aramburu J., López-Rodríguez C. (2019). Regulation of inflammatory functions of macrophages and T lymphocytes by NFAT5. Front. Immunol..

[34] Cheung C.Y.K., Ko B.C.B. (2013). NFAT5 in cellular adaptation to hypertonic stress-regulations and functional significance. J. Mol. Signal..

[35] Kumar R., Dumond J.F., Khan S.H., Thompson E.B., He Y., Burg M.B., Ferraris J.D. (2020). NFAT5, which protects against hypertonicity, is activated by that stress via structuring of its intrinsically disordered domain. Proc. Natl. Acad. Sci. USA.

[36] Laskowski T., Rezvani K. (2020). Adoptive cell therapy: Living drugs against cancer. J. Exp. Med..

[37] Redeker A., Arens R. (2016). Improving adoptive T cell therapy: The particular role of T cell costimulation, cytokines, and post-transfer vaccination. Front. Immunol..

[38] Li D., Li X., Zhou W.-L., Huang Y., Liang X., Jiang L., Yang X., Sun J., Li Z., Han W.-D. (2019). Genetically engineered T cells for cancer immunotherapy. Signal Transduct. Target. Ther..

[39] Jiang T., Zhou C., Ren S. (2016). Role of IL-2 in cancer immunotherapy. OncoImmunology.

[40] Bechman N., Maher J. (2020). Lymphodepletion strategies to potentiate adoptive T-cell immunotherapy—What are we doing; where are we going?. Expert Opin. Biol. Ther..

[41] Sharpe M., Mount N. (2015). Genetically modified T cells in cancer therapy: Opportunities and challenges. Dis. Models Mech..

[42] Zhao L., Cao Y.J. (2019). Engineered T cell therapy for cancer in the clinic. Front. Immunol..

[43] Furlan S.N., Singh K., Lopez C., Tkachev V., Hunt D.J., Hibbard J., Betz K.M., Blazar B.R., Trapnell C., Kean L.S. (2020). IL-2 enhances ex vivo–expanded regulatory T-cell persistence after adoptive transfer. Blood Adv..

[44] Wilck N., Matus M.G., Kearney S.M., Olesen S.W., Forslund K., Bartolomaeus H., Haase S., Mähler A., Balogh A., Markó L. (2017). Salt-responsive gut commensal modulates TH17 axis and disease. Nature.

[45] Ren J., Crowley S.D. (2019). Role of T-cell activation in salt-sensitive hypertension. Am. J. Physiol. Circ. Physiol..

[46] Willebrand R., Hamad I., Van Zeebroeck L., Kiss M., Bruderek K., Geuzens A., Swinnen D., Côrte-Real B.F., Markó L., Lebegge E. (2019). High salt inhibits tumor growth by enhancing anti-tumor immunity. Front. Immunol..

[47] He W., Xu J., Mu R., Li Q., Lv D.-L., Huang Z., Zhang J., Wang C., Dong L. (2020). High-salt diet inhibits tumour growth in mice via regulating myeloid-derived suppressor cell differentiation. Nat. Commun..

[48] Gerriets V.A., Kishton R.J., Johnson M.O., Cohen S., Siska P.J., Nichols A.G., Warmoes M.O., de Cubas A.A., MacIver N.J., Locasale J.W. (2016). Foxp3 and Toll-like receptor signaling balance Treg cell anabolic metabolism for suppression. Nat. Immunol..

[49] Salmond R.J. (2018). mTOR regulation of glycolytic metabolism in T cells. Front. Cell Dev. Biol..

[50] Chi H. (2012). Regulation and function of mTOR signalling in T cell fate decisions. Nat. Rev. Immunol..

[51] Saravia J., Raynor J.L., Chapman N.M., Lim S.A., Chi H. (2020). Signaling networks in immunometabolism. Cell Res..

[52] Alberdi M., Iglesias M., Tejedor S., Merino R., Lopez-Rodriguez C., Aramburu J. (2017). Context-dependent regulation of Th17-associated genes and IFNgamma expression by the transcription factor NFAT5. Immunol. Cell Biol..

[53] Kleinewietfeld M., Manzel A., Titze J., Kvakan H., Yosef N., Linker R.A., Muller D.N., Hafler D.A. (2013). Sodium chloride drives autoimmune disease by the induction of pathogenic TH17 cells. Nature.

[54] Ritz E., Koleganova N., Piecha G. (2009). Role of sodium intake in the progression of chronic kidney disease. J. Ren. Nutr..

[55] Tellechea M., Buxade M., Tejedor S., Aramburu J., Lopez-Rodriguez C. (2018). NFAT5-regulated macrophage polarization supports the proinflammatory function of macrophages and T lymphocytes. J. Immunol..

[56] Monteleone I., Marafini I., Dinallo V., Di Fusco D., Troncone E., Zorzi F., Laudisi F., Monteleone G. (2017). Sodium chloride-enriched diet enhanced inflammatory cytokine production and exacerbated experimental colitis in mice. J. Crohns Colitis.

[57] Moon E.K., Wang L.-C.S., Bekdache K., Lynn R.C., Lo A., Thorne S.H., Albelda S.M. (2018). Intra-tumoral delivery of CXCL11 via a vaccinia virus, but not by modified T cells, enhances the efficacy of adoptive T cell therapy and vaccines. OncoImmunology.

[58] Moon E.K., Carpenito C., Sun J., Wang L.C., Kapoor V., Predina J., Powell D.J., Jr Riley J.L., June C.H., Albelda S.M. (2011). Expression of a functional CCR2 receptor enhances tumor localization and tumor eradication by retargeted human T cells expressing a mesothelin-specific chimeric antibody receptor. Clin. Cancer Res..

[59] Yang J.C. (2015). Toxicities associated with adoptive T-cell transfer for cancer. Cancer J..

